# Characterization of glycoside hydrolase family 11 xylanase from *Streptomyces* sp. strain J103; its synergetic effect with acetyl xylan esterase and enhancement of enzymatic hydrolysis of lignocellulosic biomass

**DOI:** 10.1186/s12934-021-01619-x

**Published:** 2021-07-08

**Authors:** Svini Dileepa Marasinghe, Eunyoung Jo, Sachithra Amarin Hettiarachchi, Youngdeuk Lee, Tae-Yang Eom, Yehui Gang, Yoon-Hyeok Kang, Chulhong Oh

**Affiliations:** 1 Korea Institute of Ocean Science and Technology, 2670, Iljudong-ro, Gujwa-eup, Jeju, Republic of Korea; 2 Department of Ocean Science, University of Science and Technology, (34113) 217, Gajeong-ro, Yuseong-gu, Daejeon, Republic of Korea; 3 Department of Fisheries and Aquaculture, Faculty of Fisheries and Marine Sciences and Technology, University of Ruhuna, Matara, Sri Lanka

**Keywords:** Xylanase, *Streptomyces* sp. strain J103, Expression, Purification, Synergism, Lignocellulosic biomass

## Abstract

**Background:**

Xylanase-containing enzyme cocktails are used on an industrial scale to convert xylan into value-added products, as they hydrolyse the β-1,4-glycosidic linkages between xylopyranosyl residues. In the present study, we focused on *xynS1*, the glycoside hydrolase (GH) 11 xylanase gene derived from the *Streptomyces* sp. strain J103, which can mediate XynS1 protein synthesis and lignocellulosic material hydrolysis.

**Results:**

*xynS1* has an open reading frame with 693 base pairs that encodes a protein with 230 amino acids. The predicted molecular weight and isoelectric point of the protein were 24.47 kDa and 7.92, respectively. The gene was cloned into the pET-11a expression vector and expressed in *Escherichia coli* BL21(DE3). Recombinant XynS1 (rXynS1) was purified via His-tag affinity column chromatography. rXynS1 exhibited optimal activity at a pH of 5.0 and temperature of 55 °C. Thermal stability was in the temperature range of 50–55 °C. The estimated K_m_ and V_max_ values were 51.4 mg/mL and 898.2 U/mg, respectively. One millimolar of Mn^2+^ and Na^+^ ions stimulated the activity of rXynS1 by up to 209% and 122.4%, respectively, and 1 mM Co^2+^ and Ni^2+^ acted as inhibitors of the enzyme. The mixture of rXynS1, originates from *Streptomyces* sp. strain J103 and acetyl xylan esterase (AXE), originating from the marine bacterium *Ochrovirga pacifica*, enhanced the xylan degradation by 2.27-fold, compared to the activity of rXynS1 alone when Mn^2+^ was used in the reaction mixture; this reflected the ability of both enzymes to hydrolyse the xylan structure. The use of an enzyme cocktail of rXynS1, AXE, and commercial cellulase (Celluclast® 1.5 L) for the hydrolysis of lignocellulosic biomass was more effective than that of commercial cellulase alone, thereby increasing the relative activity 2.3 fold.

**Conclusion:**

The supplementation of rXynS1 with AXE enhanced the xylan degradation process via the de-esterification of acetyl groups in the xylan structure. Synergetic action of rXynS1 with commercial cellulase improved the hydrolysis of pre-treated lignocellulosic biomass; thus, rXynS1 could potentially be used in several industrial applications.

**Supplementary Information:**

The online version contains supplementary material available at 10.1186/s12934-021-01619-x.

## Background

The cost-effective and environmental-friendly utilisation of lignocellulosic biomass is a sustainable approach for biomass-based industries [1]. The production of biofuel from lignocellulosic biomass has received considerable attention as a solution to fossil fuel reserve depletion [2]. However, the complex, recalcitrant nature of lignocellulosic biomass hampers its utilisation; this is the main obstacle faced by biomass-based industries. Hemicellulose, which acts as a physical shield covering cellulose fibres, is one of the major factors responsible for this recalcitrant nature [3, 4]. It is composed of a heterogeneous mixture of xylan, xyloglucan, mannans, and glucomannans [5]; xylan was identified to be the most abundant hemicellulose in lignocellulosic biomass [6].

Xylan, a renewable bio-resource, is a major structural polysaccharide and one of the predominant hemicelluloses in plant cell walls [7]. It is comprised of a linear backbone containing β-1,4 linked d-xylopyranose residues. Short side chains of l-arabinofuranose are linked to the C-3 position of d-xylose residues and 4-*O*-methyl d-glucuronosyl groups, linked to the C-2 position of the d-xylose chain [8, 9]. Xylose is cross-linked with cellulose and lignin via covalent and non-covalent linkages [10]. Xylan can be found in two major forms in wood, i.e., as acetylated xylan and arabinoxylan in hardwood and softwood, respectively [11]. The complex and heterogenic structure of xylan is targeted by several xylolytic enzymes that exhibit synergetic activity; these enzymes include endo-1,4-β-xylanase (EC 3.2.1.8) and 1,4-β-xylosidase (EC 3.2.1.37). They act as the key enzymes that cleave the β-1,4-glycosidic linkages between xylopyranosyl residues and α-D glucuronidases (EC 3.2.1.139), α-L-arabinofuranosidase (EC 3.2.1.55), acetylxylan esterase (EC 3.1.1.72), and ferulic acid esterase (EC 3.1.1.73), for the hydrolysis of side chains (http://www.cazy.org), [12, 13]. Based on the results of amino acid sequence analysis [14] xylanases are categorised into the following glycosyl hydrolase (GH) families: 5, 7, 8, 10, 11, 43, and 52; the majority of studied xylanases belong to the GH 10 and GH 11 families (http://www.cazy.org) [12, 15].

The complex and heterogeneous properties of plant xylan and the different degrees of accessibility of xylanases into heteroxylan necessitate the identification of xylanases with different properties. Thus, numerous experiments have been performed to characterise the biochemical properties and substrate specificities of novel xylanases that can efficiently perform xylan hydrolysis. The ability of xylan to bind and hydrolyse structurally different heteroxylans is attributable to the different mechanisms of action of xylanase [15, 16]. Over the last decade, xylanase has attracted attention because it can potentially be used in various industrial processes for food, pulp, paper, and biofuel production [17, 18]. Xylanases with commercial value have reportedly been derived from marine and terrestrial bacteria, actinomycetes, and fungi [19]. It is well known that the genus *Streptomyces* is used for the production of commercially available antibiotics [20] and enzymes [21]; these organisms are recognised as being lignocellulosic [22] and chitin degraders [23]. The following evidence shows that xylanases were produced by members belonging to the genus *Streptomyces*: the *Streptomyces* sp. strain C1-3 showed the highest xylanase activity at a pH of 3.0 and temperature of 90 °C [24]; GH 11 xylanase produced by thermotolerant *Streptomyces* sp. SWU10 had a wide pH range of 0.6–10.3 [25]; xylanase derived from *Streptomyces* sp. SKK1-8 has an optimal pH and temperature of 6.0 and 50 °C, respectively; [26] and the xylanase obtained from *Streptomyces olivaceoviridis* E-86 exhibited a high activity [27]. Moreover, there are several reports regarding the production of antibiotics such as fungichromin, actinomycin X_2_, and antifungalmycin by different *Streptomyces* strains. [20, 28, 29].

In this study, a Gram-positive *Streptomyces* sp. strain J103 producing xylanase belonging to GH 11 was isolated from Incheon, South Korea. The production of xylanase from *Streptomyces* sp. strain J103 was identified through a study carried out to investigate the effective microwell plate-based screening method for microbes producing cellulases and xylanases [30]. It was used in the cloning, expression, and biochemical characterisation processes. The synergetic effect of xylanase enzyme was detected with AXE originating from marine bacteria, *O. pacifica* reported by Hettiarachchi et al. [19]. Further experiments were designed to determine the synergetic action of enzyme cocktails on pre-treated lignocellulosic biomass hydrolysis.

## Results

### Sequence analysis of x*ynS1*

**Fig. 1:**
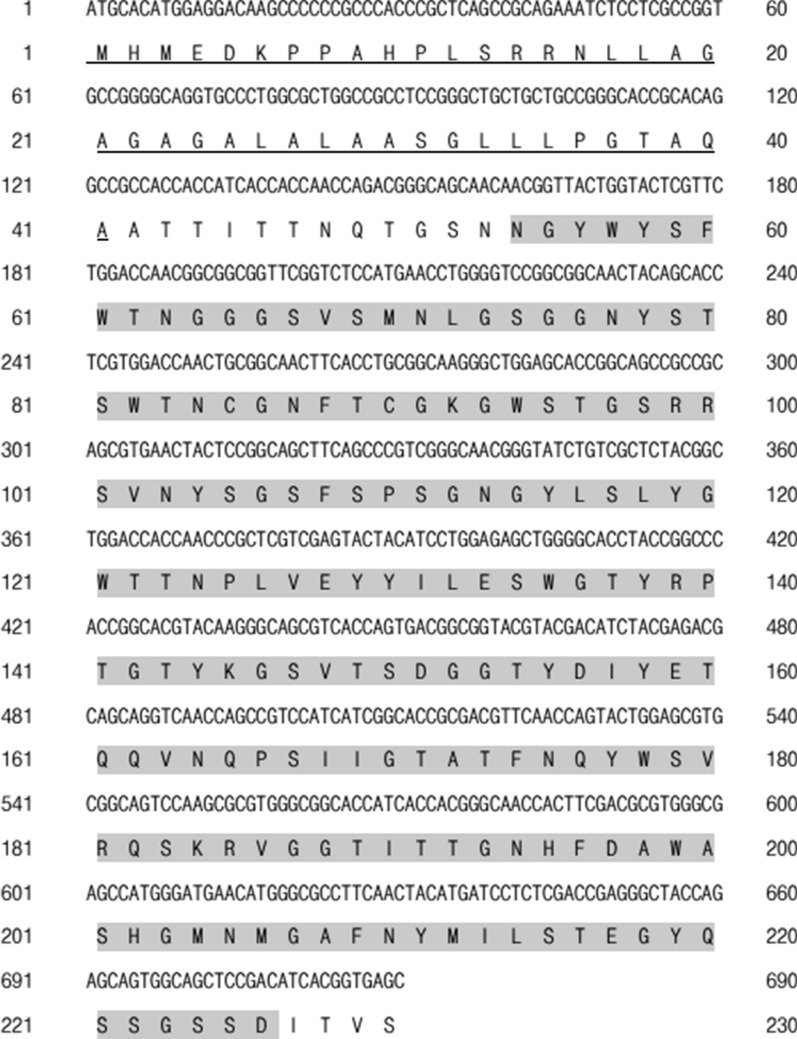
Nucleotide and amino acid sequence of XynS1. The N-terminal signal sequence (1-41aa) is underlined. Highlighted regions represent the catalytic domain of XynS1 (54–226 aa)

*xynS1* contains an open reading frame of 693 base pairs that encodes a 230 amino acid (aa) product (Fig. 1). The N-terminal region of the sequence contains a TAT signal peptide with 41 amino acids for extracellular protein secretion [31]. The signal peptide is followed by a catalytic domain comprised of GH family 11 (162–678). The predicted molecular mass and isoelectric point were 24.47 kDa and 7.92, respectively. Results of similarity and identity analysis of the XynS1 amino acid sequence obtained from *Streptomyces* sp. strain J103 revealed that XynS1 exhibited 73.2% identity and 85.7% similarity with the characterised endo-1,4-beta-xylanase precursor of *Streptomyces* sp. S38. XynS1 also shared 70.7% and 68.2% amino acid sequence identities with characterised xylanases from *Streptomyces rameus* and *Streptomyces* sp. S27 respectively (Table 1).

**Table 1:** Identity and similarity of XynS1 with uncharacterized and characterized amino acid sequences from members of the genus *Streptomyces*

Organism	Identity (%)	Similarity (%)	Gap (%)	Accession No	Remark
*S*. *griseofuscus*	99.6	99.6	0.0	RRQ74848.1	Uncharacterized
*Streptomyces* sp. SID4946	99.6	99.6	0.0	MYQ95319.1	Uncharacterized
*S. phaeogriseichromatogenes*	98.3	99.1	0.9	MBA9049746.1	Uncharacterized
*S*. *costaricanus*	97.4	98.7	0.9	MBA9057743.1	Uncharacterized
*Streptomyces* sp. S38	73.2	85.7	1.7	CAA67143.1	Characterized [32]
*S*. *rameus*	70.7	81.0	4.3	AFW21197.1	Characterized [33]
*Streptomyces* sp. S27	68.2	82.8	2.1	ACF57948.1	Characterized [34]

### Expression and purification of rXynS1

**Fig. 2:**
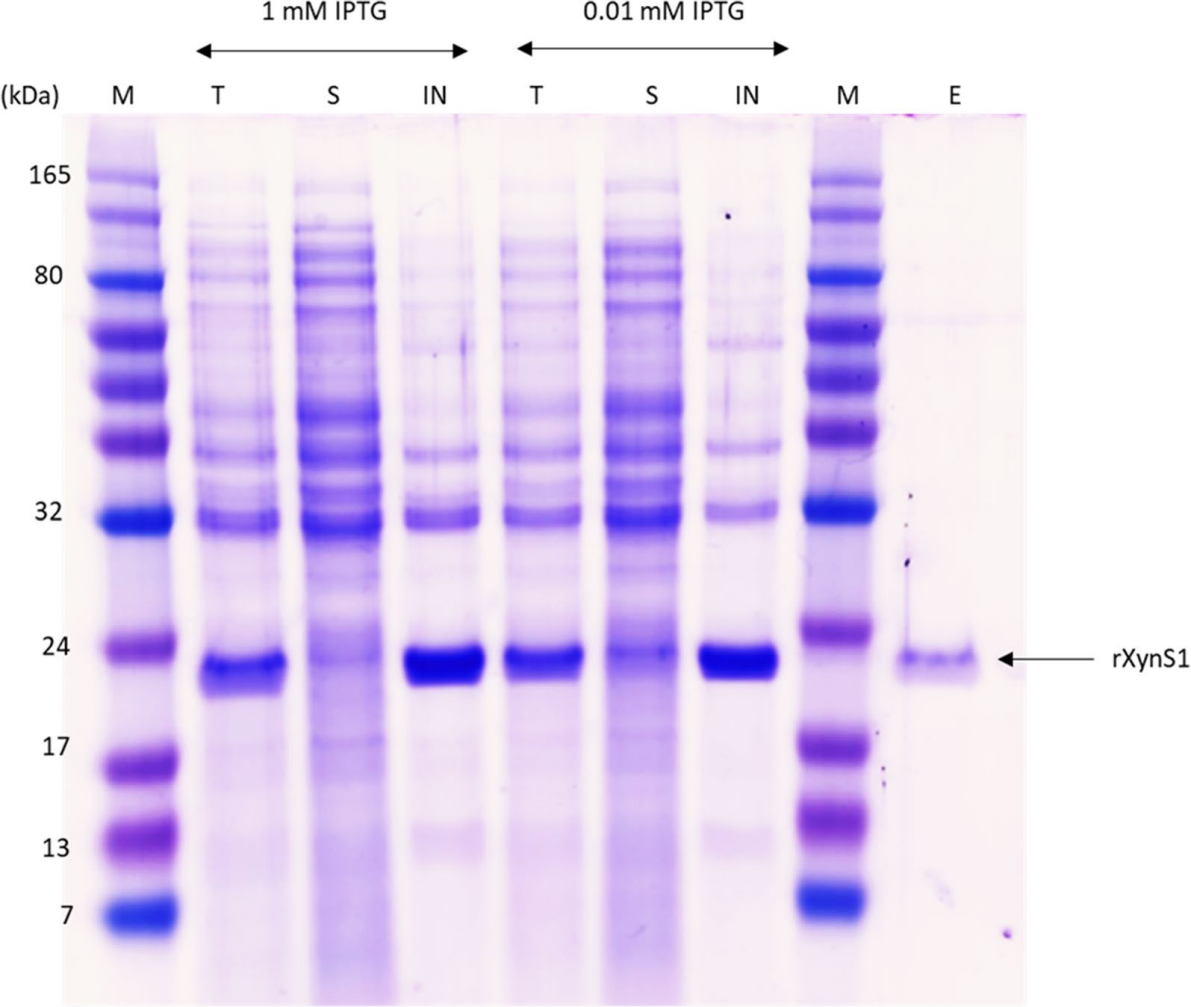
SDS-PAGE analysis of rXynS1 after induction with 1 mM and 0.01 mM IPTG. The lane abbreviations indicate the followings: M, protein marker; T, whole cell lysate after IPTG induction; S, soluble protein after IPTG induction; IN, insoluble protein after IPTG induction; E, protein purified using the His·Bind® Resin Chromatography Kit (5 times diluted)

The *xynS1* gene, which encodes endo-1,4-β-xylanase, was amplified and expressed in the pET-11a vector. In this experiment, the N-terminal signal peptide was removed, and a 6-histidine tag was attached to the C-terminal, to facilitate the affinity purification of the recombinant protein. SDS-PAGE analysis results show the presence of soluble protein after 0.01 mM IPTG induction compared with 1 mM IPTG induction (Fig. 2). The protein was purified through one-step affinity column chromatography, using the His·Bind® Resin Chromatography Kit. The molecular mass of the expressed protein in SDS-PAGE was ~ 21 kDa.

### Effect of pH and temperature on rXynS1 activity

**Fig. 3:**
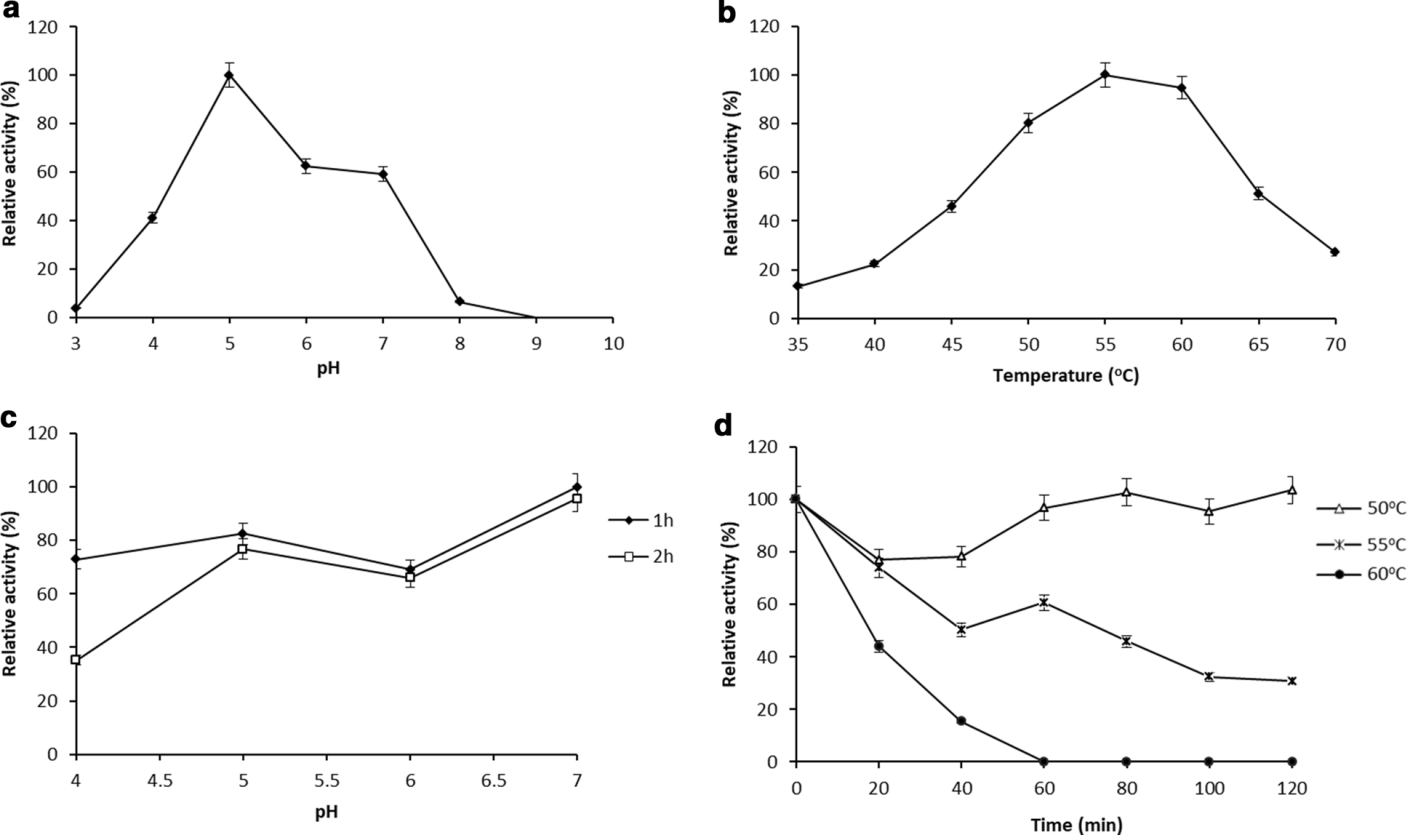
Effect of pH and temperature on rXynS1 activity. **a** pH profile for the rXynS1 enzyme. **b** Temperature profile for rXynS1. **c** Stability of rXynS1 at a pH range from 4.0 to 7.0. **d** Thermostability of rXynS1 over duration of 120 min. We used 1% beechwood xylan as the substrate in all assays. Data are shown as mean ± standard deviation (sd) values; n = 3

The effect of pH and temperature on the release of reducing sugars from beechwood xylan is shown in Fig. 3. Maximum relative activity was observed at pH 5.0, and the enzyme had a relative activity of over 58% at pH 6.0 and 7.0. Relative activities were drastically decreased for pH values below a pH of 4.0 and over a pH of 8.0 (Fig. 3a). rXynS1 showed optimal activity at 55 °C (assayed at pH 5.0), with the enzyme still displaying over 80% of its activity in the temperature range of 50–60 °C (Fig. 3b). According to the pH stability assay results, the relative activities were higher than 65% for the pH range starting from 4.0–7.0 and 5.0–7.0 after an incubation period of 1 h and 2 h, respectively (Fig. 3c). The enzyme was incubated with phosphate citrate buffer (pH 5.0) at 50 °C, 55 °C, and 60 °C (Fig. 3d) to test the thermal stability. The relative activity of rXynS1 was retained over 80% and 40% throughout the incubation period at 50 °C and 55 °C respectively, within 120 min. After a 40 min incubation period at 60 °C, the enzyme had lost its activity. Commercial xylanase (cXyl) with a concentration similar to that of rXynS1 was used to study rXynS1 activity at different pH and temperatures (see Additional file 1). cXyl exhibited an optimal pH at 7.0 and optimal temperature at 70 °C. The estimated K_m_ and V_max_ values at optimal conditions were 51.4 mg/mL and 898.2 U/mg, respectively (see Additional file 2).

### Effect of metal ions on rXynS1 activity

**Fig. 4:**
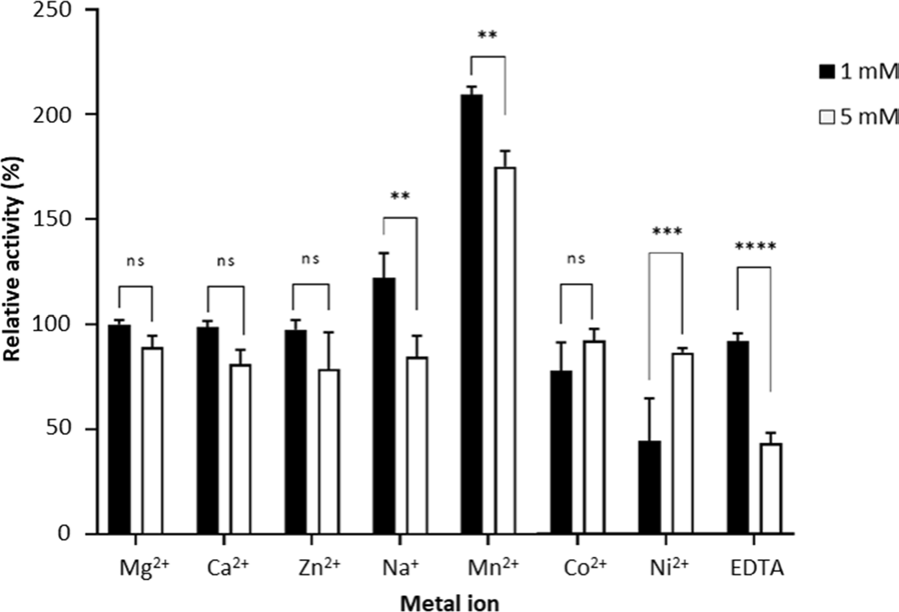
Effects of metal ions (1 mM and 5 mM) on the relative activities of rXynS1. The activity of rXynS1 in the absence of metal ion was taken as control (100%). Means with different asterisks represent the significant difference (p ≤ 0.05). ns, not significant

The association of proteins with metal ions and the formation of complexes with molecules related to the enzymes cause enzyme activation and inhibition [35]. Sanghi et al. [36] and Qiu et al. [37] reported the effect of metal ions on the enzymatic activities of xylanase from *Bacillus subtilis* ASH and GH10 xylanase obtained from frozen soil respectively. Thus we also studied the effects of various metal ions on the activity of rXynS1 using beechwood xylan as the substrate. The relative activity of the rXynS1 enzyme was stimulated by 1 mM Mn^2+^ (209.5%) and Na^+^ (122.4%). An increase in the metal ion concentration of up to 5 mM resulted in a reduction in relative activities attributable to Mn^2+^ (175.1%) and Na^+^ (88.7%). However, Mn^2+^ showed a high relative activity at a concentration of 5 mM (Fig. 4). Zn^2+^ and Ca^2+^ metal ions (1 mM and 5 mM) slightly reduced enzymatic activity but over 80% of activity was retained. Relative activity was greatly reduced to 43.4% due to the addition of 5 mM ethylenediaminetetraacetic acid (EDTA). Co^2+^ and Ni^2+^ act as inhibitors of enzyme activity. Additionally, the effect of metal ions on rXynS1 activity was measured using cXyl supplemented on a concentration basis equal to that of rXynS1 as the positive control (see Additional file 3). As similar to rXyns1, cXyl also exhibited high relative activities for reaction mixtures containing 1 mM and 5 mM Mn^2+^ and reduction in relative activity for 5 mM EDTA.

### Synergistic effect of rXynS1 with AXE

**Fig. 5:**
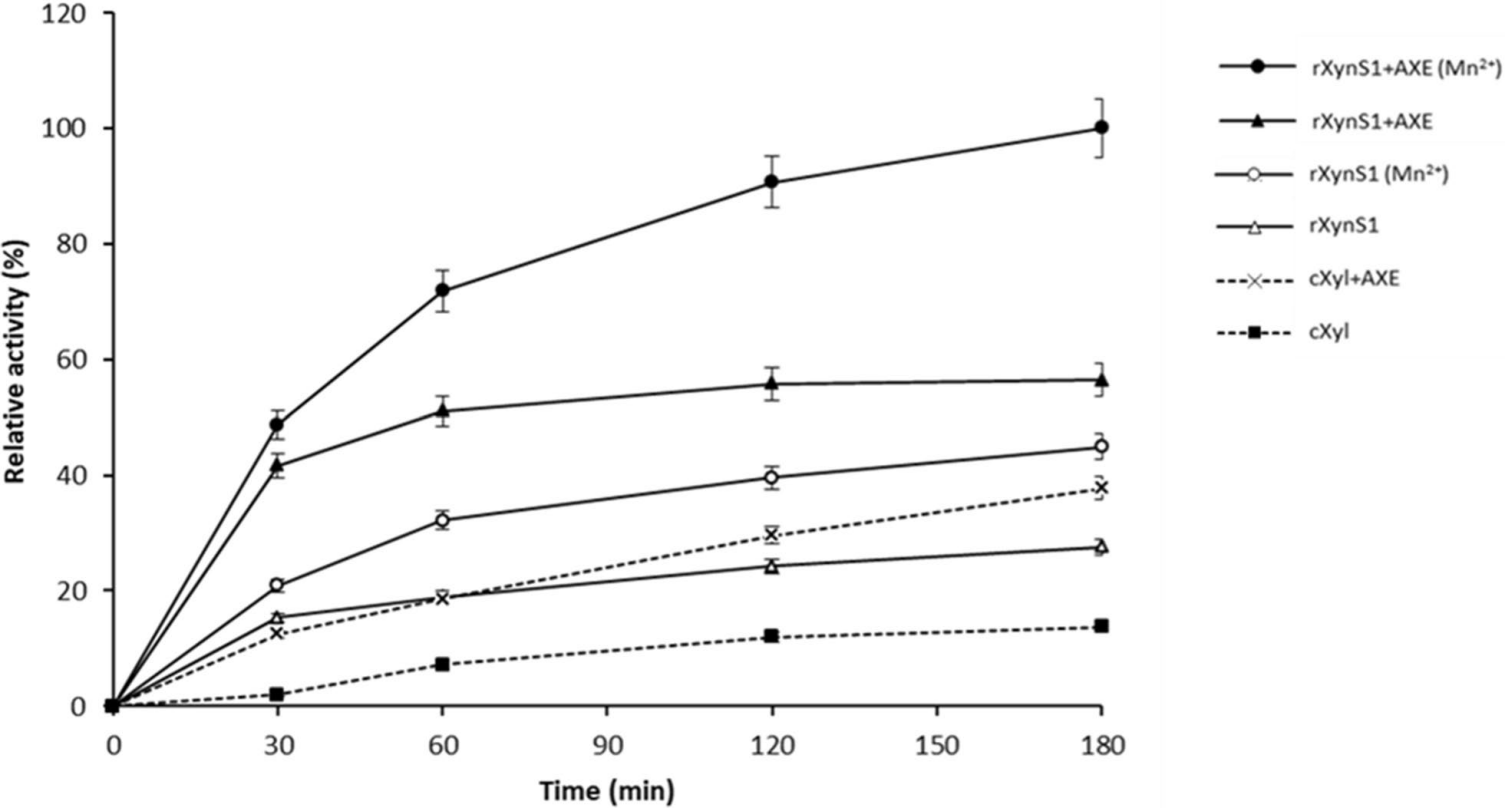
Synergetic effects of rXynS1 with AXE purified from the marine bacterium *O. pacifica*. We used 1% beechwood xylan (w/v) as the substrate in phosphate buffer (pH 7.0). Experiment was conducted using rXynS1 (Mn^2+^) and rXynS1 + AXE (Mn^2+^) by adding 1 mM Mn^2+^ into the reaction mixture and rXynS1, AXE and rXynS1 + AXE without adding metal ions into the reaction mixture. Incubation was performed for 180 min at a temperature of 50 °C. Data are shown as mean ± standard deviation (sd) values; n = 3

Several studies on the synergism between xylanases and acetyl xylan esterases reported by Hettiarachchi et al. and Yang et al. [19, 38] showed the removal of acetyl groups in xylan and improvement in xylan hydrolysis. Experiments were performed to investigate the synergetic effect of XynS1 derived from *Streptomyces* sp. strain J103 and AXE derived from the marine bacteria *O. pacifica* on xylan hydrolysis (Fig. 5). Beechwood xylan (1%) (w/v) was used as the substrate. Experiment was conducted using reaction mixtures containing 1 mM Mn^2+^ and a mixture without metal ions to investigate the effect of synergetic activity of rXynS1 with AXE in hydrolyzing 1% beechwood xylan (w/v). Commercially available xylanase (cXyl) was used as the positive control. Relative activity was not observed when only AXE without metal ions was used in the reaction mixture under standard conditions. The cocktail of rXynS1 + AXE (Mn^2+^) enhances the xylan hydrolysis by 2.27 and 1.90-folds than that of rXynS1 (Mn^2+^) and rXynS1 + AXE without metal ions respectively. According to the experimental data, the combination of rXynS1 and AXE enhanced the xylan hydrolysis than rXynS1 alone with and without metal ions. rXynS1 + AXE and rXynS1 increased their relative activities by 1.49 and 2.0-folds than that of cXyl + AXE and cXyl respectively, when the same amount of commercial xylanase (cXyl) was used as that of rXynS1.

### Synergistic effect of rXynS1, AXE, and commercial cellulase on the hydrolysis of lignocellulosic biomass

**Fig. 6:**
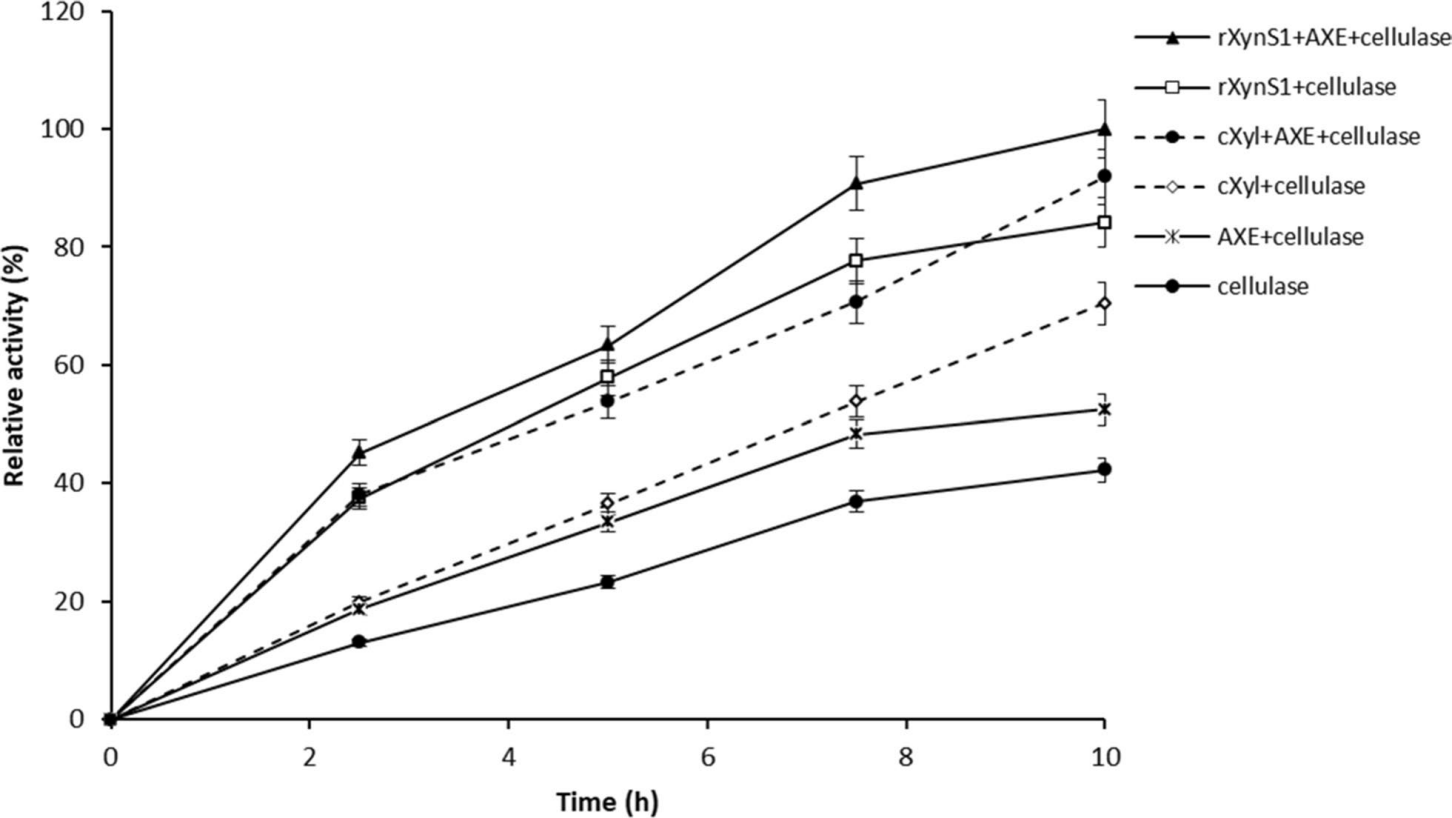
Effect of enzyme cocktails on hydrolysis of pre-treated lignocellulosic biomass. Pre-treated biomass was incubated with enzymes for appropriate time intervals at 50 °C. The substrate to which no enzyme was added was used as the control

Lignocelluloses are the most abundant plant-derived biomass which are composed of celluloses, hemicelluloses, and lignin. Chemical factors such as lignin, hemicellulose, acetyl groups, and physical factors such as crystallinity and particle size influence lignocellulosic biomass recalcitrance, which makes it difficult to achieve lignocellulose hydrolysis [5]. Zhang et al. [39] reported that the cellulose hydrolysis of lignocellulose biomass was inhibited in the presence of xylan. Most of the hemicelluloses derived from straw or grass are made up of xylan [40]. Study conducted by Selig et al. [41] revealed the combination of xylanase, acetyl xylan esterase and cellulolytic enzyme as an effective combination in enhancing the synergistic improvements in cellulose conversion. Thus we also performed an experiment using enzyme cocktails prepared with the rXynS1 derived from *Streptomyces* sp. strain J103, AXE derived from *O. pacifica*, commercial xylanase (cXyl), and commercial cellulase Celluclast® 1.5 L on determining lignocellulosic biomass degradation capability. Biomass pre-treatment was performed to eliminate the lignin wrapped around the cellulose and hemicellulose structure, thereby increasing the accessibility of cellulases and other enzymes towards the cellulose and hemicellulose structure [42]. After 10 h of enzymatic hydrolysis of pre-treated lignocellulose biomass, the enzyme cocktails comprising rXynS1 + AXE + cellulase and rXynS1 + cellulase increased their relative activities by 1.08 and 1.19-folds than cXyl + AXE + cellulase and cXyl + cellulase respectively. Figure 6 shows that the enzyme combination rXynS1 + AXE + cellulase was significantly more effective than AXE + cellulase and commercial cellulase alone. After performing hydrolysis for 10 h, the enzyme combination rXynS1 + AXE + cellulase enhanced lignocellulosic biomass degradation by 1.18, 1.90 and 2.36-folds than that of rXynS1 + cellulase, AXE + cellulase and commercial cellulase alone. These results suggest that the addition of rXynS1 significantly increased the cellulose hydrolysis of lignocellulosic biomass, as the hydrolysis of hemicellulose increased the accessibility of enzymes toward cellulose.

## Discussion

In the present study, the *Streptomyces* sp. strain J103 was isolated from Incheon, South Korea. *Streptomyces* sp. strain J103 was screened for xylanase activity via the 3, 5- dinitrosalicylic acid (DNS) method, using xylose as the standard. We successfully cloned the xylanase gene into pET-11a and expressed it in *E. coli* BL21(DE3). The xylanase enzyme, XynS1, belongs to the GH Family 11, which consists of true endo-β-1,4-xylanases that cleave internal β-1,4-glycosidic bonds [43]. Generally, GH 11 xylanases consist of a single catalytic domain with a β-jelly roll architecture and have two antiparallel β-sheets, A and B, with a long and deep cleft [44].

According to the results of the conserved domain analysis performed using NCBI, XynS1 encodes a 230 amino acid product and contains an N-terminal signal peptide with 41 amino acids, followed by a catalytic domain. Most of the secretory proteins in genus *Streptomyce*s are directly released to the culture supernatant [45]. Thus to avoid disruptions in protein production, the experiments were performed in the absence of an N-terminal signal peptide containing TAT signal. Xylanase enzyme was produced by *Streptomyces* sp. strain J103 after an incubation period of 72 h at 15 °C and optimal enzyme assay conditions were determined. A production yield of 630 μg/L rXynS1 was obtained from 1L culture media. The predicted molecular mass and isoelectric point were 24.47 kDa and 7.92, respectively. According to Wong et al. [11], xylanases can be categorized into two families, based on the correlation between their molecular weight and pI: members with a high MW (> 30 kDa) and acidic pI were classified into family F and members with a low MW (< 30 kDa) and basic pI were grouped in family G. Thus, XynS1 can be classified into family G, but this system cannot be applied to all newly discovered xylanases [44]. The substrate of xylanases, xylan, is found in the cell walls of land plants and some alga, including red and green algae, with a variety of structures [46, 47]. Beechwood xylan was used as the substrate for enzymatic assay analysis. rXynS1 exhibits optimal activity at pH 5.0, and high relative activity at pH 6.0 and 7.0. Joshi et al. [48] demonstrated that the optimal pH values of GH 11 xylanases are correlated with the nature of the residue adjacent to the acid/base catalyst. Substitution of an aspartic acid for an asparagine residue at position 35 results in a pronounced change in the pH optimum of *Bacillus circulans* xylanase from pH 5.7 to 4.6 and a slight increase in activity [48]. According to the study of Torronen and Rouvinen [49], xylanases with more acidic pH optimum are placed with aspartic acid adjacent to the acid/base catalyst, whereas it is asparagine in alkaline xylanases. rXynS1 functions at an optimum temperature of 55 °C, but still retains approximately 80% of its activity at 50–60 °C. Xylanases such as endo-1,4-beta-xylanase *Cfl Xyn11A* from *Cellulomonas flavigenas* showed the optimal temperature at 55 °C as similar to rXynS1 [50]. Most of the studied GH11 xylanases exhibits their optimal temperature at 50 °C which is comparable with rXynS1 i.e., endo-beta-1,4-xylanase from *Bacillus licheniformis* strain I5 [51] and xylanase from *Cellvibrio mixtus* strain J3-8 xylanases [52]. But there are also some thermophilic xylanases with optimum functioning at high temperatures. Xylanases derived from *Streptomyces* sp. strain C1-3 [24] and *Bacillus thermantarcticus* [53] exhibited optimum temperatures at 90 °C and 80 °C respectively. The thermal stability of the enzyme at 50 °C resulted in a constant relative activity of over 90% for 60 min after an incubation period of 1 h. The decrease in xylanase activity at an optimal temperature during the incubation period is believed to result from the change in the three-dimensional structure of the enzyme due to thermal denaturation. Hydrogen, non-covalent bonds, ionic, and van der Waals bonds are interrupted by heat and cause changes in the native protein structure [54].

One millimolar of Mg^2+^, Na^+^ and Mn^2+^ stimulated enzyme activity up to 100.1%, 122.4% and 209.5%, respectively. According to the previous study by Sanghi et al. [36] the relative activity of xylanase derived from *Bacillus subtilis* ASH, increased up to 185% in the presence of 1 mM Mn^2+^ which is comparable with the relative activity of rXynS1 in presence of 1 mM Mn^2+^ (209.5%). In contrast to rXynS1, Mg^2+^ and Na^+^ decreased the relative enzyme activities of xylanase derived from *Bacillus subtilis* ASH up to 84.5% and 88.5%. A study by Park et al. [55] also reported a xylanase derived from *Paenibacillus* sp. KIJ1 enhancing the enzyme activity by 5 mM Mn^2+^. According to the results of the current study, relative enzyme activity of rXynS1 decreased in the presence of 1 mM and 5 mM Ca^2+^ and Zn^2+^ metal ions. Besides, 1 mM Ca^2+^ and Zn^2+^ slightly inhibited the activity of rXynS1 (98.8% and 97.5%) than 5 mM Ca^2+^ and Zn^2+^ (81.3% and 79.1%). But the study carried out by Qiu et al. [37] showed an enhancement of relative enzyme activity of a cold active xylanase, Xyn27 up to 129.1% and 106.4% in the presence of 5 mM Ca^2+^ and Zn^2+^. Although the use of 5 mM Mn^2+^ resulted in decreased xylanase activity, compared to that observed with a concentration of 1 mM, the relative enzyme activities were enhanced (Fig. 4). Co^2+^, Ni^2+^, and EDTA inhibited xylanase activity. Interestingly, the relative xylanase activities resulting from the use of 1 mM Co^2+^ and Ni^2+^ are lower than those observed with a metal ion concentration of 5 mM. Riordan et al. [35] described the presence of different types of interactions between metals, enzymes, and substrates. The typical behavior of metal-activated enzymes is the interaction between metal and substrate before the formation of the enzyme–substrate complex. In the second type, metal first binds to the enzyme before the enzyme–substrate complex formation. Thirdly, metal acts on a site away from the active site of the enzyme either to maintain the protein structure or to regulate the catalytic activity. Interactions of Ni^2+^ with sulfhydryl groups of the enzyme change the protein confirmation and affect the enzyme inactivation [56]. Moreover the metal ion interactions with sulfhydryl groups, carboxyl groups also may affect the enzyme activity by altering the protein conformation [57]. The chelating agent EDTA readily inactivated the enzyme at a concentration of 5 mM. High level of enzyme activity observed with the addition of 1 mM EDTA, which can be attributed to the fact that EDTA renders the substrate more accessible to the enzyme by chelating certain ions linked to the enzyme [58]. When the EDTA concentration exceeds a certain level (5 mM), the enzyme is inactivated. A study carried out by Kerovuo et al. [58] showed that the complete inactivation of *B. subtilis* phytase occurred upon the elimination of the metal ion requirement of the enzyme using EDTA.

AXE derived from the marine bacteria *O. pacifica* was used to investigate the synergetic effect with rXynS1 on xylan hydrolysis. According to the results of conserved domain analysis, the AXE derived from *O. pacifica* has been classified under the alpha/beta hydrolase family [19]. Mn^2+^ was used in the experiment as Mn^2+^ can improve the enzymatic activities of rXynS1 and AXE. According to the experimental results, the combination of rXynS1 and AXE enhanced the xylan hydrolysis than rXynS1 alone with and without metal ions. The synergetic effect of rXynS1 + AXE (Mn^2+^) and rXynS1 + AXE without metal ions enhanced the beechwood xylan degradation by 2.27 and 2.05 respectively than rXynS1 (Mn^2+^) and rXynS1 without metal ions alone. The relative activity of commercial xylanase (cXyl) was less than that of rXynS1 alone. Due to the addition of AXE, the relative activity of cXyl was enhanced by 1.37-fold than that of rXynS1 alone. AXE increases the accessibility of xylanase enzymes to the substrates, as most of the acetyl groups present in the xylan chain can hinder xylanase activity [59]. Thus, it is important to perform deacetylation efficiently, to increase the hydrolysis yield of xylan and produce non-acetylated hydrolysis products [60]. The study carried out by Kim et al. [61] showed the synergetic effect of acetyl xylan esterase from *Lactobacillus antri* DSM 16,041 and *Thermotoga neapolitana* β-xylanase enhancing the beechwood xylan degradation by 1.44-fold higher than xylanase alone. Hettiarachchi et al. [19] also observed the synergistic effect of xylanase and AXE on enhancement of xylan hydrolysis by 1.41-fold than xylanase alone during the xylan degradation. Zhang et al. [62] mentioned that the activity of xylanase derived from *Plectosphaerella cucumerina* was reduced by 36.5% after adding 5 mM Mn^2+^. According to the results of this study, the presence of 1 mM Mn^2+^ in the reaction mixture containing both rXynS1 and AXE enhanced its activity than the reaction mixtures with other enzyme combinations. This kind of phenomenon created by Mn^2+^ is advantageous in industrial applications since metal ions are inescapable factor in industrial processes.

Wheat straw is considered to be one of the main crop residues worldwide [6]. Thus, it is cost-effective to use wheat straw in biomass industries for biofuel production; this would address the fossil fuel crisis via the conversion of lignocellulosic biomass to value-added products. One of the drawbacks in the commercialization of lignocellulosic biofuels is the high cost of enzymes. Extensive research has been carried out to determine the best enzyme or enzyme cocktails for efficient biomass hydrolysis [63]. We also performed an experiment to study the synergistic effect of enzyme cocktails on the hydrolysis of pre-treated lignocellulosic biomass using rXynS1. According to the experimental results, the enzyme cocktail comprised of rXynS1 + AXE + cellulase, cXyl + AXE + cellulase and rXynS1 + cellulase showed high relative activities (2.36, 2.17 and 1.99-fold increase), compared to that observed with cellulase alone. The relative activity of reaction mixture containing rXynS1 + AXE + cellulase enhanced the lignocellulosic hydrolysis by 1.18-fold than the mixture containing rXynS1 + cellulase. After performing the hydrolysis for 10 h, enzyme combination rXynS1 + AXE + cellulase enhanced lignocellulosic biomass degradation by 1.90-fold than that of AXE + cellulase, suggesting the effect caused by rXynS1 is much higher than effect caused by AXE. Hemicelluloses act as a physical barrier limiting enzyme accessibility. Thus, the removal of hemicellulose, and especially the xylan and acetyl groups, could enhance cellulose hydrolysis [64]. A study was carried out by Zhang et al. [60] to identify the synergy between different xylanolytic enzymes and cellulases in the hydrolysis of the pretreated wheat straw. They revealed that the supplementation of cellulases with xylanolytic enzymes increased the hydrolysis degree of substrates compared with cellulases alone. Thus, rXynS1 represents a possible candidate for industrial applications, which could effectively hydrolyse xylan and lignocellulose biomass.

## Conclusion

The complete hydrolysis of xylan, a major hemicellulose component, requires the synergetic action of an array of enzymes. The conversion of xylan into useful products and production of cost-efficient xylanases with desired characteristics is necessary to meet industrial application-related requirements. Thus, the identification of new enzymes that can be used in industrial processes is necessary. In the present study, we have reported about a xylanase derived from the *Streptomyces* sp. strain J103 that belongs to the GH 11 family. We cloned and expressed the relevant gene in the pET-11a expression vector, and used molecular techniques to improve the biochemical characteristics and expression levels of the enzyme. We also studied the synergetic effects of the enzyme with the acetyl xylan esterase derived from *O. pacifica*, and designed experiments to determine the extent of synergism of xylanase in an enzyme cocktail, in order to understand the hydrolysis of the pre-treated lignocellulosic biomass. It is important to understand enzymes and their proportions more effectively, in order to design efficient enzyme cocktails for lignocellulose degradation. An approach involving microbes improved via genetic engineering could be used with enzyme technology techniques to identify less expensive and effective enzyme systems.

## Methods

### Identification and molecular characterization of xylanase

We had previously isolated a xylanase producing *Streptomyces* sp. strain J103 [30] and analysed its genome sequence. A putative xylanase sequence was predicted and designated as *xynS1.* The conserved domains of XynS1 were predicted using the National Center for Biotechnology Information (NCBI) Conserved Domain Database (CDD; https://www.ncbi.nlm.nih.gov/cdd/). The Signal IP-5.0 server (http://www.cbs.dtu.dk/services/SignalP/) [65] was used to predict the N-terminal signal peptide of the xylanase amino acid sequence. The isoelectric point and molecular weight were determined using the DNADynamo software (Blue Tractor Software, North Wales, UK). The closest neighbour proteins of the XynS1 amino acid sequence were recognised using NCBI BLAST (https://blast.ncbi.nlm.nih.gov) and used to determine the identity, similarity, and gap percentages against the XynS1 amino acid sequence using the EMBOSS Pairwise Sequence Alignment Tool (https://www.ebi.ac.uk/Tools/psa/) [66]. The amino acid sequence of xylanase was submitted to Gene Bank under accession number MW357597.

### Cloning of the *xynS1*gene

Genomic DNA was extracted using the Accuprep Genomic DNA Extraction Kit (Bioneer, Daejon, South Korea). The primers via polymerase chain reaction (PCR) amplified the xylanase gene without the signal peptide. The xylanase forward primer sequence (GAGACATATGGCCACCACCATCACCACCAA), which included the *Nde* I site, and the reverse primer sequence (GAGAGGATCCTCAATGATGATGATGATGATGGCTCACCGTGATGTCGGAGCTG), which included 6 histidine tag, stop codon, and *Bam*H I, were synthesised (underlined bases resembling the restriction site). The PCR mixture consisted of 200 ng of genomic DNA, 25 μL of 2X PrimeSTAR GC buffer (Takara Bio Inc., Shiga, Japan), forward and reverse primers (20 pmol each), 4 μL of 2.5 mM dNTP (Takara Bio Inc., Shiga, Japan), and 2.5 units of PrimeSTAR HS DNA polymerase (Takara Bio Inc., Shiga, Japan). The PCR process included an initial denaturation step at 94 °C for 5 min and 30 cycles of reactions per cycle with a denaturation, annealing, and extension step at 94 °C for 30 s, 48 °C for 30 s, and 72 °C for 1 min, respectively, and a final extension step at 72 °C for 5 min. Confirmed PCR products in agarose gel were purified using the Accuprep Gel Purification kit (Bioneer, Daejon, South Korea). The purified PCR products and pET-11a vector were digested with *Nde* I and *Bam*H I restriction enzymes (Takara Bio Inc., Shiga, Japan), according to the manufacturer’s instructions. The digested PCR product was ligated into the digested pET-11a vector (Novagen, Madison, USA) using T4 DNA ligase (Takara Bio Inc., Shiga, Japan), and the recombinant plasmid was transformed into *E. coli* DH5α cells, by subjecting them to a heat shock. The positive clone was selected and cultured in 5 mL of Luria–Bertani (LB) broth supplemented with ampicillin (100 ng/μL) at 37 °C and an agitating speed of 180 rpm overnight. Plasmid DNA was extracted and purified using the Accuprep Nano-plus Plasmid Mini Extraction Kit (Bioneer, Daejon, South Korea). After verifying the extracted DNA sequence, recombinant plasmids were transformed into the expression host *E. coli* BL21(DE3) via the heat shock method.

### Expression and purification of rXynS1

The transformed *E. coli* BL21(DE3) cells were cultured in LB broth containing ampicillin (100 ng/μL) at 37 °C overnight at an agitation speed of 180 rpm and 12 ml of overnight culture was used to inoculate 500 mL LB broth containing ampicillin (100 ng/μL). Culture media was induced using isopropyl-β-D-thiogalactopyranoside (IPTG) to obtain a final concentration of 0.01 mM at an optical density and wavelength of 0.5 and 600 nm, respectively. The broth media containing host cells were incubated at 15 °C and 180 rpm for 72 h. Cells were harvested via centrifugation at a speed of 2000 × g for 30 min and resuspended in the buffer recommended by the user protocol of His·Bind® Resin Chromatography Kit (Novagen, San Diego, CA, USA). Cells were disrupted by sonication and the supernatant was collected by performing centrifugation at 13,000 × g at 4 °C for 30 min. The supernatant was passed through an affinity column containing Ni–NTA resin. Purification was performed with the His·Bind® Resin Chromatography Kit. The xylanase protein containing the His-tag was recovered in an elution buffer containing 0.5 mM NaCl, 20 mM Tris HCl, and 1 M imidazole. The concentration of the protein was calculated using the Pierce™ BCA Protein Assay Kit (Thermo Fisher Scientific, Daejeon, South Korea), according to the manufacturer’ s protocol [67]. Using 12% sodium dodecyl sulphate polyacrylamide gel electrophoresis (SDS-PAGE), the molecular weight and purity of the protein were determined. The Pink Prestained Protein Marker (NIPPON Genetics EUROPE) was used as a reference.

### Enzyme activity assay

The activity of rXynS1 was detected using DNS (Sigma-Aldrich, St Louis, MO, USA) method [68], a colorimetric method for measuring the amount of reducing sugars released from the substrate. We used 1% beechwood xylan (w/v) (Tokyo Chemical Industry Co. Ltd., Tokyo, Japan) as the substrate. A reaction mixture of 195 μL containing 1% beechwood xylan (w/v) and 5 μL of the enzyme with a concentration of 36 μg/mL was incubated at 55 °C for 10 min. The activity of the enzyme was terminated by incubating the mixture of enzyme and substrate with the DNS solution at 95 °C for 10 min. Xylose (Sigma-Aldrich, St Louis, MO, USA) was used as the standard. Absorbance was measured at a wavelength of 575 nm to detect the amount of xylose released within 10 min. One unit of xylanase activity was defined as the amount of enzyme that yielded 1 µmol of reducing sugars within 1 min of reaction.

### Biochemical characterization of purified enzyme

The optimal pH for enzyme activity was measured using 1% beechwood xylan (w/v) dissolved in the following different buffer solutions: phosphate citrate buffer (pH 3.0–5.0), phosphate buffer (pH 6.0–8.0), and glycine–NaOH buffer (pH 9.0–10.0), under standard assay conditions. The pH stability was determined by incubating the enzyme in different pH buffers at 24 °C for 1 h and 2 h, and the relative activity was measured under standard assay conditions. The optimal temperature for xylanase activity was determined by incubating 1% beechwood xylan (w/v) over a temperature range of 35–70 °C with an interval of 10 min at 5 °C in phosphate citrate buffer (pH 5.0). To determine its thermostability, the diluted enzyme was incubated at temperatures of 50 °C, 55 °C, and 60 °C in the absence of a substrate over time periods of 0, 20, 40, 80, 100, and 120 min. Activity tests were performed under standard assay conditions, after cooling the preincubated enzyme samples on ice for 5 min. The non-pretreated enzyme was used as the control. The effect of metal ions on xylanase activity was measured using 1% beechwood xylan (w/v) as the substrate. Solutions containing different metal ions, i.e., Ca^2+^, Mg^2+^, Co^2+^, Mn^2+^, Zn^2+^, Na^+^, Ni^2+^, and EDTA were dissolved in buffer solutions to result in a final concentration of 1 mM and 5 mM. The activity was measured under standard assay conditions. A reaction mixture without metal ions was used as the control. Statistical analysis was performed using GraphPad Prism, version 9.0.1 (GraphPad Software, Inc, USA). Additionally, commercial xylanase (cXyl) (Sigma Aldrich: X2753) (Sigma-Aldrich, St Louis, MO, USA) derived from *Thermomyces lanuginosus* (expressed in *Aspergillus oryzae*) was used on a concentration basis equal to that of rXynS1 to compare the effect of pH and temperature on rXynS1 activity and the effect of metal ions on rXynS1 activity under standard assay conditions. K_m_ and V_max_ values were calculated at optimal conditions in presence of 1 mM Mn^2+^ using the Michaelis-Menton equation using GraphPad Prism, version 9.0.1. Beechwood xylan (5–15 mg/mL) was used as the substrate.

### Synergistic effect of rXynS1 with AXE

Experiments were carried out with 1% beechwood xylan (w/v) in phosphate buffer (pH 7.0). AXE from the marine bacterium *O. pacifica* was used to investigate the synergetic activity of rXynS1. AXE was expressed and purified using the His·Bind® Resin Chromatography Kit, according to the methodology described by Hettiarachchi et al. [19]. rXynS1 (0.18 μg of purified protein/1.6 U), AXE (0.05 U) and cXyl (with a same quantity used as rXynS1) were used in the experiment. Experiment was performed using reaction mixtures containing 1 mM Mn^2+^ and a reaction mixture without metal ions. Experiment was conducted using rXynS1 (Mn^2+^) and rXynS1 + AXE (Mn^2+^) by adding 1 mM Mn^2+^ in to the reaction mixture and rXynS1, AXE and rXynS1 + AXE without adding metal ions in to the reaction mixture. Experiment was further conducted with reaction mixture containing cXyl and cXyl + AXE without adding metal ions. A reaction mixture (200 μL) containing 1% beechwood xylan (w/v) in phosphate buffer (pH 7.0) was used. Incubation was carried out at 50 °C and the activity was measured for 3 h using DNS assay.

### Synergistic effect of rXynS1, AXE, and commercial cellulase on the hydrolysis of lignocellulosic biomass

Wheat straw was obtained from the local market and washed thoroughly to remove any debris. The dried wheat straw was ground into a powder and pre-treated with 1.5% NaOH at a ratio of 1:10 (w/v) at 70 °C for 4 h. The pre-treated mass was filtered and washed until its pH was neutral. One percent pre-treated wheat straw in phosphate citrate buffer (pH 6.0) was used as the substrate. rXynS1 (0.11 μg of purified protein/1 U), AXE (0.03 U), cXyl (with a same quantity used as rXynS1) and a commercially available cellulase, Celluclast® 1.5 L (1.4 U) (Novozymes A/S, Bagsvaerd, Denmark) derived from *Trichoderma reesei* were used in the experiment. The experiment was performed using a mixture of rXynS1 + AXE + cellulase, rXynS1 + cellulase, cXyl + AXE + cellulase, cXyl + cellulase, AXE + cellulase, and cellulase alone. Reactions were performed in Eppendorf tubes containing 1 mL of substrate, and the reaction mixtures were incubated at different time intervals of 0, 2.5, 5, 7.5, and 10 h at 50 °C with a speed of 150 rpm (MODEL:VS-8480, VISION SCIENTIFIC CO.,LTD, South Korea) in a shaking incubator. Samples were centrifuged at 13,000×g for 25 min. The supernatant was used to examine the reducing sugars via the DNS method. Glucose (Sigma-Aldrich, St Louis, MO, USA) was used as the standard for this experiment.

## Supplementary Information

**Additional file 1. **Effect of pH and temperature on rXynS1 activity. a. pH profile for the rXynS1 enzyme. b. Temperature profile for rXynS1. c. Stability of rXynS1 at a pH ranges from 4.0 to 7.0. d. Thermostability of rXynS1 over duration of 120 minutes. We used 1% beechwood xylan as the substrate in all assays. cXyl supplemented on a concentration basis equal to that of rXynS1 was used as the positive control. Optimum activity of cXyl in each experiment was set as 100%. Data are shown as mean ± standard deviation (sd) values; n = 3.

**Additional file 2. **Michaelis–Menten plot of rXynS1 using beechwood xylan as the substrate. The K_m_ and V_max_ values were calculated by fitting the initial enzyme activity data to a Michaelis–Menten kinetic model. The measures were performed in optimal pH and temperature in presence of 1 mM Mn^2+^ in reaction medium.

**Additional file 3. **Effects of metal ions (1 mM and 5 mM) on rXynS1 activity. The activity of cXyl supplemented on a concentration basis equal to that of rXynS1 in the absence of metal ion was taken as the positive control (100%). Means with different asterisks represent the significant difference (p ≤ 0.05). ns, not significant.

## Data Availability

All data generated or analyzed during this study are included in this published article.

## Abbreviations

GH: Glycoside hydrolase
*xynS1*: Xylanase gene
XynS1: Xylanase protein
rXynS1: Recombinant xylanase
AXE: Acetyl xylan esterase
PCR: Polymerase chain reaction
LB: Luria–Bertani
IPTG: Isopropyl-β-d-thiogalactopyranoside
SDS-PAGE: Sodium dodecyl sulfate polyacrylamide gel electrophoresis
DNS: 3, 5- Dinitrosalicylic acid
EDTA: Ethylenediaminetetraacetic acid
cXyl: Commercial xylanase
