# Supplementary material for: Characterization of glycoside hydrolase family 11 xylanase from Streptomyces sp. strain J103; its synergetic effect with acetyl xylan esterase and enhancement of enzymatic hydrolysis of lignocellulosic biomass
Source: Microb Cell Fact. 2021 Jul 8;20:129. doi: 10.1186/s12934-021-01619-x (PMC8265113; doi:10.1186/s12934-021-01619-x)
Supplement: Supplementary file 1 — Additional file 1. Effect of pH and temperature on rXynS1 activity. a. pH profile for the rXynS1 enzyme. b. Temperature profile for rXynS1. c. Stability of rXynS1 at a pH ranges from 4.0 to 7.0. d. Thermostability of rXynS1 over duration of 120 minutes. We used 1% beechwood xylan as the substrate in all assays. cXyl supplemented on a concentration basis equal to that of rXynS1 was used as the positive control. Optimum activity of cXyl in each experiment was set as 100%. Data are shown as mean ± standard deviation (sd) values; n = 3. [file 12934_2021_1619_MOESM1_ESM.docx]

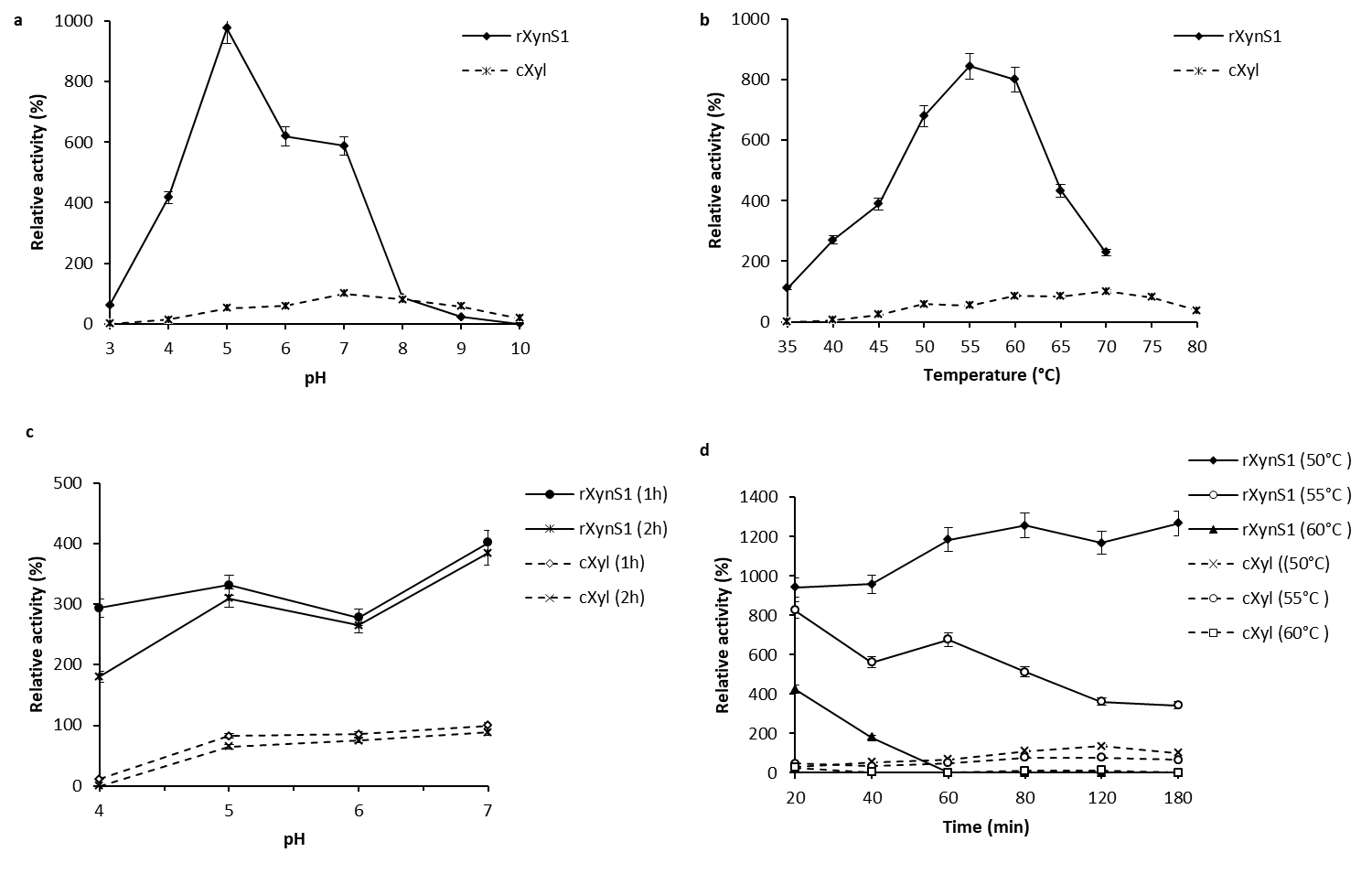


**Additional file 1. Effect of pH and temperature on rXynS1 activity.** a. pH profile for the rXynS1 enzyme. b. Temperature profile for rXynS1. c. Stability of rXynS1 at a pH ranges from 4.0-7.0. d. Thermostability of rXynS1 over duration of 120 minutes. We used 1% beechwood xylan as the substrate in all assays. cXyl supplemented on a concentration basis equal to that of rXynS1 was used as the positive control. Optimum activity of cXyl in each experiment was set as 100%. Data are shown as mean ± standard deviation (sd) values; n=3.
