# Supplementary material for: Characterization of glycoside hydrolase family 11 xylanase from Streptomyces sp. strain J103; its synergetic effect with acetyl xylan esterase and enhancement of enzymatic hydrolysis of lignocellulosic biomass
Source: Microb Cell Fact. 2021 Jul 8;20:129. doi: 10.1186/s12934-021-01619-x (PMC8265113; doi:10.1186/s12934-021-01619-x)
Supplement: Supplementary file 2 — Additional file 2. Michaelis–Menten plot of rXynS1 using beechwood xylan as the substrate. The Km and Vmax values were calculated by fitting the initial enzyme activity data to a Michaelis–Menten kinetic model. The measures were performed in optimal pH and temperature in presence of 1 mM Mn2+ in reaction medium. [file 12934_2021_1619_MOESM2_ESM.docx]

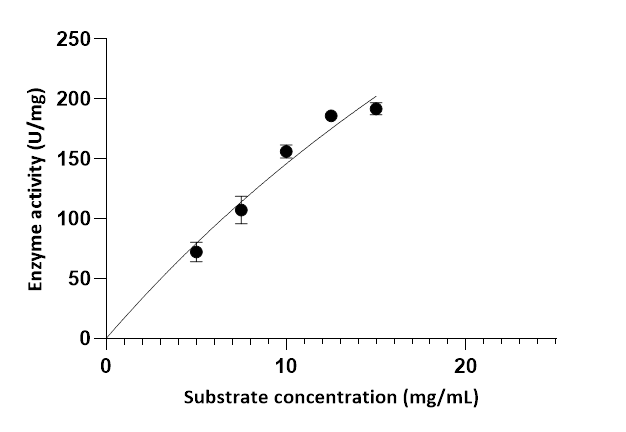


**Additional file 2. Michaelis–Menten plot of rXynS1 using beechwood xylan as the substrate.** The K_m_ and V_max_ values were calculated by fitting the initial enzyme activity data to a Michaelis–Menten kinetic model. The measures were performed in optimal pH and temperature in presence of 1 mM Mn^2+^ in reaction medium.
