# Supplementary material for: Characterization of glycoside hydrolase family 11 xylanase from Streptomyces sp. strain J103; its synergetic effect with acetyl xylan esterase and enhancement of enzymatic hydrolysis of lignocellulosic biomass
Source: Microb Cell Fact. 2021 Jul 8;20:129. doi: 10.1186/s12934-021-01619-x (PMC8265113; doi:10.1186/s12934-021-01619-x)
Supplement: Supplementary file 3 — Additional file 3. Effects of metal ions (1 mM and 5 mM) on rXynS1 activity. The activity of cXyl supplemented on a concentration basis equal to that of rXynS1 in the absence of metal ion was taken as the positive control (100%). Means with different asterisks represent the significant difference (p ≤ 0.05). ns, not significant. [file 12934_2021_1619_MOESM3_ESM.docx]

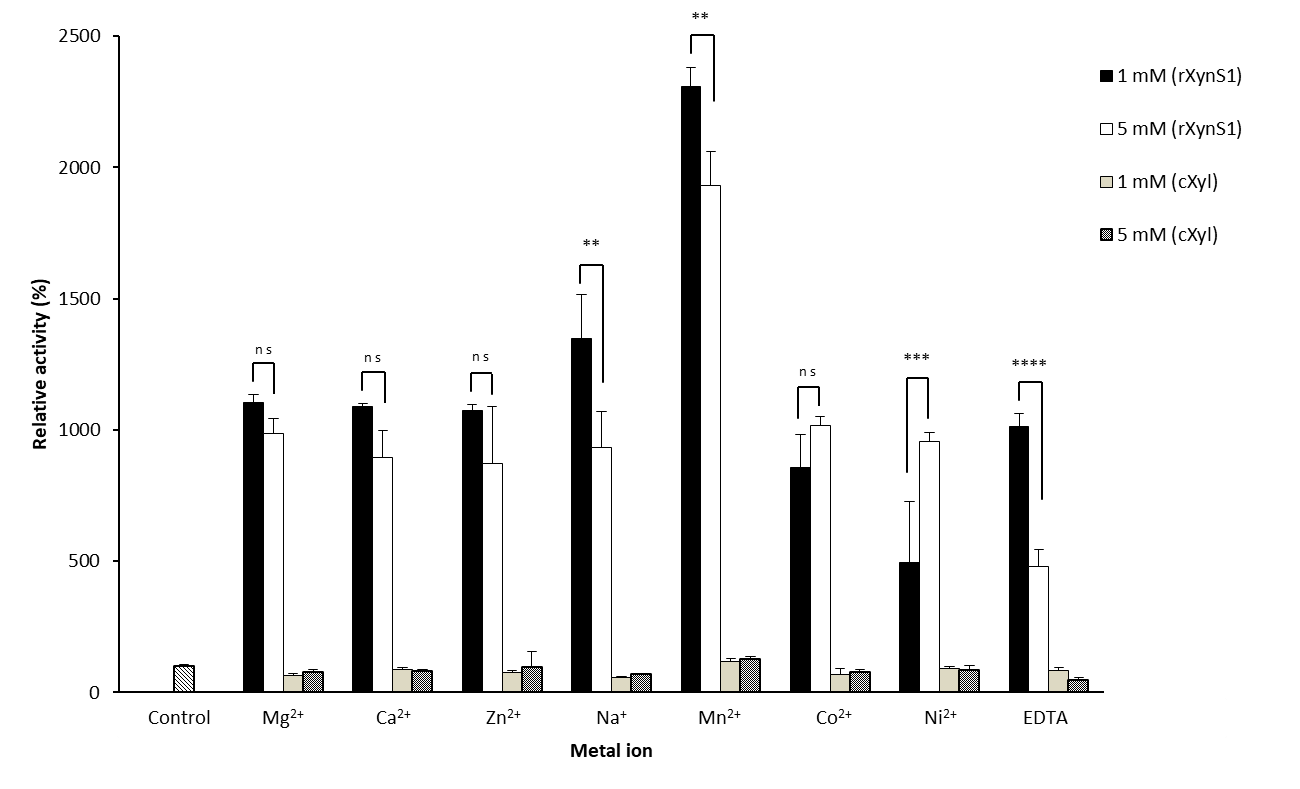


**Additional file 3. Effects of metal ions (1 mM and 5 mM) on rXynS1 activity.** The activity of cXyl supplemented on a concentration basis equal to that of rXynS1 in the absence of metal ion was taken as the positive control (100%). Means with different asterisks represent the significant difference (p ≤ 0.05). ns, not significant
